# Role of B lymphocytes in the infarcted mass in patients with acute myocardial infarction

**DOI:** 10.1042/BSR20203413

**Published:** 2021-02-03

**Authors:** Ana C.A. Casarotti, Daniela Teixeira, Ieda M. Longo-Maugeri, Mayari E. Ishimura, Maria E.R. Coste, Henrique T. Bianco, Flavio T. Moreira, Amanda F. Bacchin, Maria C. Izar, Iran Gonçalves, Adriano Caixeta, Gilberto Szarf, Ibraim M. Pinto, Francisco A. Fonseca

**Affiliations:** 1 Universidade Federal de São Paulo, São Paulo, Brazil; 2 Instituto Dante Pazzanese de Cardiologia, São Paulo, Brazil

**Keywords:** B Lymphocytes, cardiac MRI, cytokines, myocardial infarction

## Abstract

Despite early reperfusion, patients with ST segment elevation myocardial infarction (STEMI) may present large myocardial necrosis and significant impairment of ventricular function. The present study aimed to evaluate the role of subtypes of B lymphocytes and related cytokines in the infarcted mass and left ventricular ejection fraction obtained by cardiac magnetic resonance imaging performed after 30 days of STEMI. This prospective study included 120 subjects with STEMI submitted to pharmacoinvasive strategy. Blood samples were collected in subjects in the first (D1) and 30th (D30) days post STEMI. The amount of CD11b+ B1 lymphocytes (cells/ml) at D1 were related to the infarcted mass (rho = 0.43; *P*=0.033), measured by cardiac MRI at D30. These B1 cells were associated with CD4+ T lymphocytes at D1 and D30, while B2 classic lymphocytes at day 30 were related to left ventricular ejection fraction (LVEF). Higher titers of circulating IL-4 and IL-10 were observed at D30 versus D1 (*P*=0.013 and *P*<0.001, respectively). Titers of IL-6 at D1 were associated with infarcted mass (rho = 0.41, *P*<0.001) and inversely related to LVEF (rho = −0.38, *P*<0.001). After multiple linear regression analysis, high-sensitivity troponin T and IL-6 collected at day 1 were independent predictors of infarcted mass and, at day 30, only HDL-C. Regarding LVEF, high-sensitivity troponin T and high-sensitivity C-reactive protein were independent predictors at day 1, and B2 classic lymphocytes, at day 30. In subjects with STEMI, despite early reperfusion, the amount of infarcted mass and ventricular performance were related to inflammatory responses triggered by circulating B lymphocytes.

## Introduction

Almost 20% of patients with acute myocardial infarction (MI) develop heart failure, even when early reperfused [1]. Left ventricular remodeling seems related to the size of myocardial infarction and timely reperfusion, as well as to the inflammatory responses and residual ischemia [2].

Experimental studies suggested that B lymphocytes may influence the myocardial infarcted mass [3], although there are few data about the role of these cells in humans. Furthermore, a possible atheroprotective role for B1 lymphocytes has been proposed based on the production of interleukin 10 (IL-10) and natural antibodies, which may switch the proinflammatory response to more appropriate healing, promoting cell recovery and the clearance of apoptotic cellular debris [4]. On the other hand, classic B lymphocytes or B2 cells are linked to progression of atherosclerosis, possibly by their interaction with CD4+ T lymphocytes [4].

**Figure 1:**
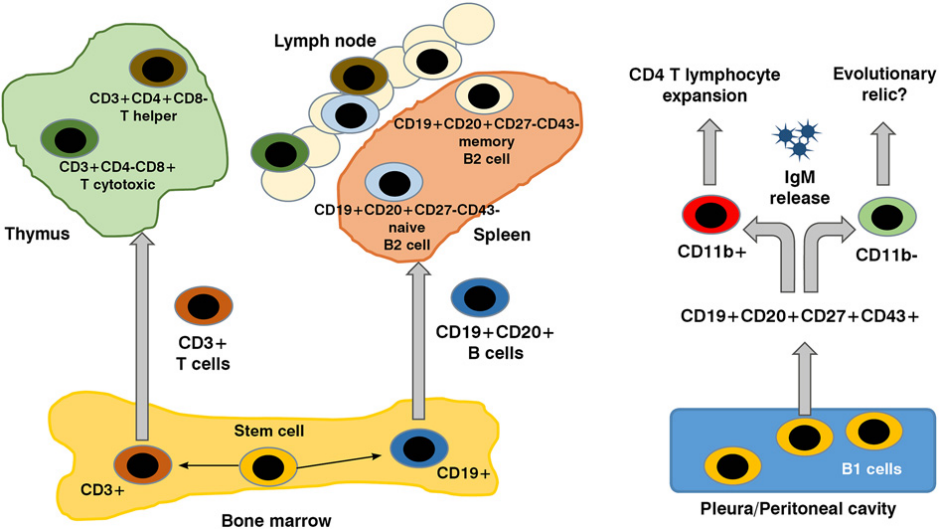
B and T cells, origin and differentiation Stem cells in blood marrow differentiate in T or B lymphocyte, according to the presence of CD3 or CD19, respectively. Lymphocyte final maturation takes place in thymus for T cells; or in spleen and lymphatic tissue for classic B cells. B1 lymphocytes are well described in experimental studies. These cells are notorious for their capacity of spontaneous production of IgM and according to the presence of the CD11b, two distinct subtypes are recognized: CD11b- B1 lymphocytes, producing IgM, and CD11b+ B1 lymphocytes, related to the expansion of CD4+ T lymphocytes.

In 2011, Griffin and colleagues proposed CD19+CD20+CD43+CD70- lymphocyte cells as the human B1 phenotype, and these cells spontaneously produced IgM and IL-10 [5]. However, according to the presence or absence of the CD11b on the surface of these cells, the capacity of IgM production and activation of CD4+ T lymphocytes can differ [6–8]. The differentiation cycle of B and T lymphocytes subpopulations is briefly shown in Figure 1.

Herein, we investigated the role of lymphocyte subpopulations, circulating interleukins and natural antibodies at baseline and after 30 days of ST segment elevation myocardial infarction (STEMI), examining their association with parameters of cardiac MRI, particularly the myocardial infarction size and left ventricular function.

## Patients and methods

### Study design and participants

**Figure 2:**
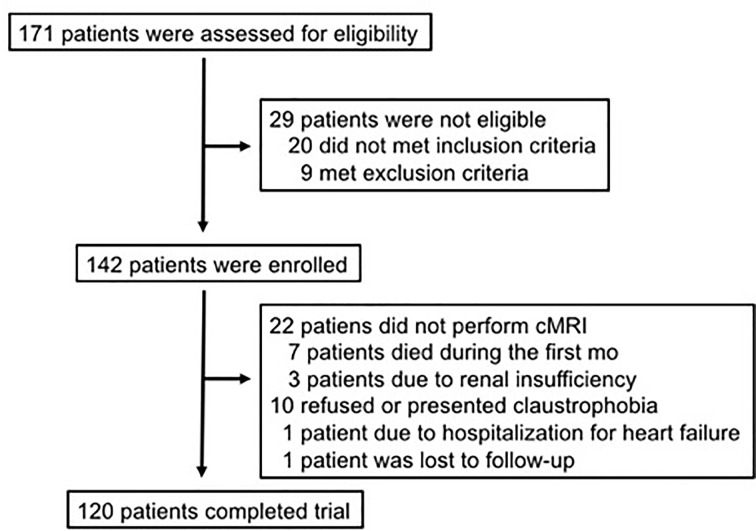
Enrollment and patient flow Of the 171 patients who were assessed for eligibility, 29 were excluded due to inclusion or exclusion criteria. A total of 152 met all inclusion criteria, but 22 did not perform cMRI due to a variety of reasons (renal insufficiency, hospitalization for heart failure, death, claustrophobia or even for refusing the exam).

For the present study, 120 consecutive adults of both sexes with their first myocardial infarction were included in the B And T Types of Lymphocytes Evaluation in Acute Myocardial Infarction (BATTLE-AMI study, NCT02428374) [9]. Figure 2 shows the total number of patients assessed, enrolled and that completed the trial. Patients treated by primary PCI were not included. All patients were submitted to pharmacological thrombolysis in the first 6 h followed by coronary angiogram and percutaneous intervention when needed, in the first 24 h of STEMI (pharmacoinvasive strategy) in Hospital Sao Paulo, a tertiary universitary hospital, in the city of Sao Paulo. According to the study protocol, all patients received standard of care therapies (including highly-effective lipid lowering drugs and dual antiplatelet strategy). These patients were randomized to receive rosuvastatin 20 mg qd (Crestor®, AstraZeneca) or simvastatin 40 mg plus ezetimibe 10 mg qd (Vytorin®, MSD) and subsequently to ticagrelor 90 mg bid (Brilinta®, Astra Zeneca) or clopidogrel 75 mg qd (Plavix®, Sanofi Aventis), in a two-by-two factorial design. The recruitment of these patients started on May 2015, and was completed on March 2020. All laboratory analysis was performed in the Hospital Sao Paulo, as well as all the coronary angiograms and percutaneous intervention. Cardiac magnetic resonance imaging (cMRI) was performed in the Instituto Dante Pazzanese de Cardiologia or in the Hospital Sao Paulo.

Key exclusion criteria were clinical instability, use of immunosuppressant therapies, autoimmune disease, known malignancy, pregnancy or signs of active infections.

The study protocol was approved by the local ethics committee (IRB 0297/2014), which follows the Declaration of Helsinki, and the written informed consent was provided by all subjects before their inclusion.

### Laboratory assays and flow cytometer analysis

Blood samples (20 ml) were collected in the first (D1) and 30th (D30) days after STEMI, and were promptly examined by flow cytometry and *in vitro* assays. All patients were transfered to our hospital after thrombolysis for coronary angiography during the 24 h of symptoms onset and blood samples were collected in the first day of hospitalization, except for those patients transfered during the night. In this case, samples were collected in the early morning of the next day.

**Figure 3:**
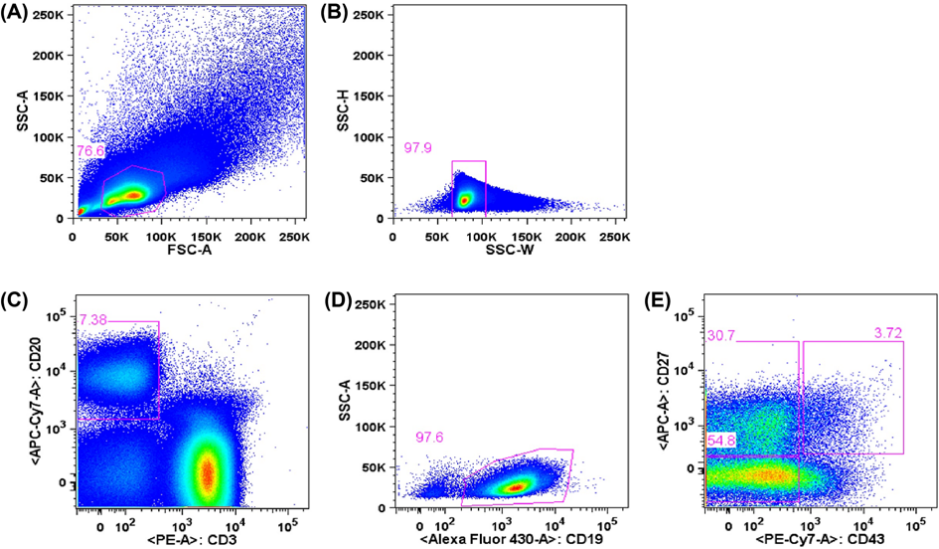
Analysis and cell sorting of B lymphocytes from peripheral blood by flow cytometry Lymphocytes were defined in a graph of size and complexity (**A**) (FSC-A x SSC-A) and analyzed as singlets (**B**) (SSC-W x SSC-H). After excluding CD3+ cells and selecting CD19+ (**C**) (CD3 x CD19) and CD20+ cells (**D**) (CD20 x SSC), B cell subpopulations were defined (**E**) (CD43 x CD27) as naïve (CD43- CD27-), memory (CD43- CD27+) and B1 cells (CD43+ CD27+). This scheme is representative of all analyzed samples. About 2,000,000 total events were acquired, and the entire sample was submitted to cell sorting.

**Figure 4:**
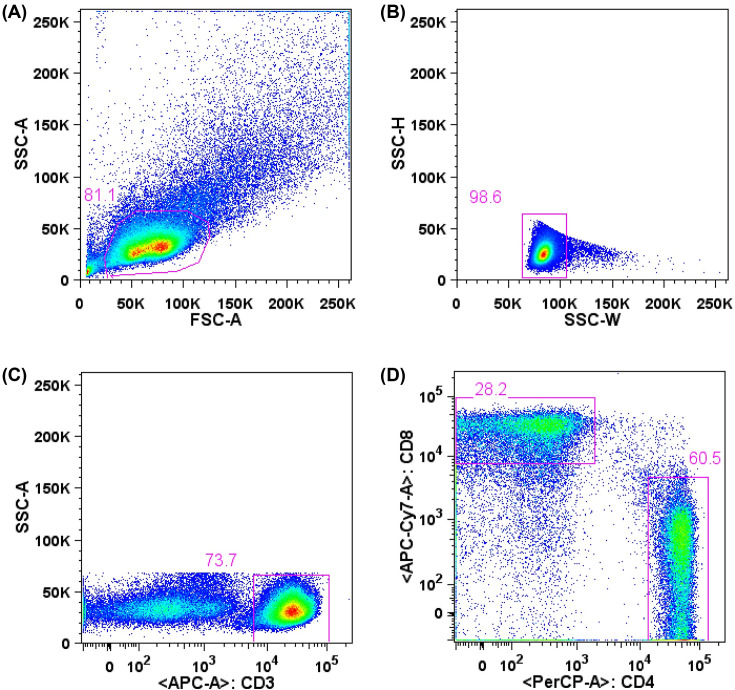
Analysis of T lymphocytes from peripheral blood by flow cytometry Lymphocytes were defined in a graph of size and complexity (**A**) (FSC-A x SSC-A) and analyzed as singlets (**B**) (SSC-W x SSC-H). Among CD3+ cells (**C**) (CD3 x SSC-A), cells were defined as exclusively CD4+ and CD8+ (**D**) (CD4 x CD8). This scheme is representative of all analyzed samples. About 50,000 CD3+ events were acquired.

The cells were thawed, diluted in RPMI medium (RPMI-1640 supplemented with 2 mM L-glutamine, 1 mM sodium pyruvate, 1% vitamin solution, 1% nonessential amino acids, penicillin/streptomycin, 28 mM HEPES, 23.8 mM sodium bicarbonate and 55 mM 2-mercaptoethanol, Gibco). After resuspending in RPMI medium supplemented with 10% of FBS (R10), viability and cell concentration were determined by trypan blue (Gibco) staining and counting in Neubauer chamber. Obtained PBMC were submitted to surface molecules staining with fluorescent-conjugated monoclonal antibodies for evaluation of T and B lymphocytes. Cells were labeled with CD19-BV510 (clone SJ25C1), CD27-APC (clone M-T271), CD20-APC-H7 (clone 2H7), CD43-PE-Cy7 (clone 1G10), CD3-PE (clone UCHT1), CD11b/MAC-1-BV421 (clone ICRF44) (BD Biosciences) and CD284 (TLR4)-Alexa Fluor^®^ 488 (clone HTA125) (eBioscience) anti-human monoclonal antibodies for evaluation of B lymphocytes, and with CD3-APC (clone SK7), CD4-PE-Cy5.5 (clone SK3) and CD8-APC-eFluor®780 (clone RPA-T8) (eBioscience), for evaluation of T lymphocytes. After washing, cells were submitted to acquisition and cell sorting using a FACSAria™ II (BD Biosciences) cytometer with appropriate compensation controls (single stained beads, BD CompBeads). B1 lymphocyte population, defined as CD3^−^ CD19^+^ CD20^+^ CD27^+^ CD43^+^, as previously described [5,6], were isolated, collected in RPMI medium, and further submitted to culture assays. Cell populations were analyzed using FlowJo Software (9.7.6 version, Tree Star). B1 cells were also analyzed for expression of CD11b surface marker. B2 lymphocyte subtypes were considered such as CD3^−^ CD19^+^ CD20^+^CD43^−^ (Figure 3). T cells analysis were defined as CD4^+^ (CD3^+^ CD4^+^ CD8^−^) and CD8^+^ (CD3^+^ CD4^−^ CD8^+^) lymphocytes (Figure 4).

The frequency of spontaneously secreting IgM cells was determined as previously described.^5^ The number of spots per well was counted using an ELISpot plate reader (Elviflo, AID) and the software ELISpot 6.0.

### Interleukin-6 in cultured T lymphocytes

T lymphocytes obtained by flow-cytometry were cultured for 3 days (50,000 cells with 100 µl/well of R10) and stimulated by phytohemagglutinin at a concentration of 5 µg/ml. After this period, the supernatant was collected and kept at −20°C. IL-6 was quantified using kit BD CBA Human Th1/Th2, using the flow-cytometer BD FACSCantoll^™^. The concentrations detected were defined by standard curve for the cytokine analyzed using the software BD FACPArray™.

### Evaluation of immunoglobulin M and circulating interleukins

For determination of IgM titers in plasma, 96-well plates were coated with 100 μl/well of purified anti-human IgM (Southern Biotech), diluted 1:8000 in 0.05 M carbonate buffer. Detection was performed after washes with PBST and 2 h of incubation with HRP-conjugated anti-human IgM (Southern Biotech) diluted 1:8,000 in blocking solution.

For the determination of circulating interleukins, enzyme-linked immunosorbent assay (ELISA) was performed using Human IL-4 ELISA Set, Human IL-6 ELISA Set and Human IL-10 ELISA Set (BD OptEIA^TM^ - BD Pharmingen-U.S.A.), according to manufacturer’s instructions.

### Cardiac magnetic resonance imaging

Cardiac MRI was performed at the 30th day after STEMI as previously reported [9], based on previous cMRI sequencial studies since baseline, reporting changes, at 30 and 180 days, in the strain of the peri-infarction myocardial or in the infarcted mass, and to explore effects of inflammatory variables during the recovery and healing of myocardial infarction [10–12]. Briefly, images were performed on 3.0 T scanners and protocol included steady-state free precession imaging (SSFP) for anatomical evaluation, retrospective cine imaging in long and short axis views and late contrast-enhanced imaging using gadolinium for evaluation of myocardium scar/fibrosis. Cine images were obtained in the two-chamber, four-chamber, left ventricular outflow tract, and short-axis views with the first slice positioned at the mitral valve and last covering the apex, resulting in 10–12 cine breath-hold short-axis images to cover the entire ventricle. Infarcted mass was quantified using myocardial delayed enhancement technique, after the injection of gadolinium-based contrast agent. Contrast-enhanced images were acquired in the same views as those used for cine MRI, with the use of a segmented inversion-recovery sequence. Each patient study was reviewed by two independent blinded readers using dedicated software. The necrotic tissue mass was estimated in grams and as the percentage of the necrotic tissue of the left ventricular mass.

### Statistical analysis

Categorical variables were compared by Pearson’s Chi-square test. Continuous variables are expressed as median (interquartile range [IQR]) or mean ± SD, and normality was tested by the Kolmogorov–Smirnov test. Paired Student’s *t*-test or the nonparametric Wilcoxon rank sum test were used to compare continuous variables at D1 and D30 after STEMI. Correlation between variables were tested by the Spearman’s rank test. Multiple linear regression analysis was performed to identify independent predictors for infarcted mass and LVEF. Sample size was estimated on basis of pilot study of our group. Statistical analysis was performed using SPSS Statistics 17.0 and significance was set at *P*<0.05.

## Results

### Patients

Baseline clinical, laboratory, and angiographic characteristics of the study population are shown in Table 1. Complete follow up was obtained in 99% of participants. At day 30, all patients were taking highly effective lipid lowering therapy and dual antiplatelet therapy. In addition, 95% were taking β-blockers, 98% angiotensin-converting enzyme inhibitors or angiotensin-receptor blockers, 22% metformin, 13% sulphonylurea and 3% insulin.

**Table 1:** Characteristics of study population at day 1 and day 30

Parameters	Day 1 (*n*=120)	Day 30 (*n*=120)	*P* value
Age (years), mean ± SD	55 ± 8	NA	
Male gender, *n* (%)	84 (70)	NA	
Smokers, *n* (%)	48 (40)	NA	
Hypertensives, *n* (%)	72 (60)	NA	
Diabetes, *n* (%)	34 (28)	NA	
Cholesterol, mg/dl, median (IQR)	203 (176–243)	127 (107–153)	<0.001
LDL-cholesterol, mg/dl, median (IQR)	133 (109–163)	65 (43–86)	<0.001
HDL-cholesterol, mg/dl, median (IQR)	42 (34–49)	38 (32–44)	0.107
Triglycerides, mg/dl, median (IQR)	133 (93–215)	131 (104–161)	0.068
Non HDL-C, mg/dl, median (IQR)	165 (135–205)	92 (67–116)	<0.001
HbA1c (%), median (IQR)	5.9 (5.6–6.6)	NA	
hs-TNT, ng/l, median (IQR)	4140 (1563–9915)	NA	
Creatinine, mg/dl, median (IQR)	0.84 (0.7–1.02)	0.93 (0.81–1.08)	<0.001
GFR, ml/min/m^2^, median (IQR)	91 (79–100)	88 (71–98)	0.001
hsCRP, mg/l, median (IQR)	16.1 (7.1–36.8)	2.3 (1.0–7.4)	<0.001
Culprit coronary artery			
Left anterior descending, *n* (%)	52 (43)	NA	
Right coronary artery, *n* (%)	48 (40)	NA	
Left Circumflex artery, *n* (%)	15 (13)	NA	
MINOCA, *n* (%)	5 (4)	NA	
SBP, mm Hg, median (IQR)	130 (110–140)	118 (108–130)	<0.001
DBP, mm Hg, median (IQR)	80 (70–90)	71 (65–80)	<0.001

Abbreviations: DBP, diastolic blood pressure; GFR, glomerular filtration rate (CKD-EPI); hsCRP, high-sensitivity C-Reactive protein; hs-TNT, high-sensitivity troponin T; MINOCA, myocardial infarction with nonobstructive coronary artery; SBP, systolic blood pressure. Comparisons made by Wilcoxon test; NA, not applicable.

### Biochemical parameters

Total cholesterol, LDL-C, and non-HDL-C were decreased at day 30 (all *P*<0.001 vs. day 1). No significant changes were observed for triglycerides or HDL-C. There was an increase in serum creatinine levels, and a decrease in high-sensitivity C-reactive protein (hsCRP) levels and glomerular filtration rate (GFR). All comparisons were made by the Wilcoxon test (Table 1).

### Leukocytes, lymphocytes and immunoglobulin M (IgM)

Leukocytes and lymphocytes (cells/ml) at D30 were lower than at D1. There was a significant decrease in B lymphocytes subtypes and in CD4+ T lymphocytes at D30 compared with D1 (Table 2).

**Table 2:** Leukocytes and subpopulations of lymphocytes after myocardial infarction

Leukocytes	1st day, *n*=120	30th day, *n*=120	*P* value
WBC count	10855 (9160–13375)	7620 (6568–8928)	<0.001
Total lymphocyte count	2195 (1522–2994)	1973 (1542–2431)	0.001
T Lymphocytes			
CD4^+^ (CD3^+^CD4^+^)	921 (627–1274)	886 (697–1155)	0.049
CD8^+^ (CD3^+^CD8^+^)	339 (212–501)	324 (195–504)	0.097
B Lymphocytes			
B2 (CD19^+^CD20^+^CD43^−^)	144 (87–229)	108 (68–175)	<0.001
B1 (CD19^+^CD20^+^CD27^+^CD43^+^)	5.25 (3.03–10.71)	3.82 (2.21–7.12)	<0.001
B1 CD11b^+^	1.75 (1.02–3.45)	1.41(0.68–2.29)	<0.001
B1 CD11b^−^	3.33 (1.61–6.67)	2.19 (1.20–4.77)	<0.001

WBC, white blood cells. Values are cells/ml expressed by median and interquartile range. Data compared by Wilcoxon test.

We found an association between CD11b+ B1 cells and CD4+ T lymphocytes at D1 (rho = 0.34, *P*<0.001) and at D30 (and rho = 0.29, *P*=0.001).

Higher circulating titers of IgM were observed at D30 (*P*<0.001 vs. D1), and they were related to B1 cells (rho = 0.22, *P*=0.014). These correlations were also observed for subtypes of B1 cells. Spontaneously secreted IgM was observed in the supernatant of cultured CD11b+ and CD11b- B1 cells at D1 and D30.

### Circulating interleukins (IL)

**Figure 5:**
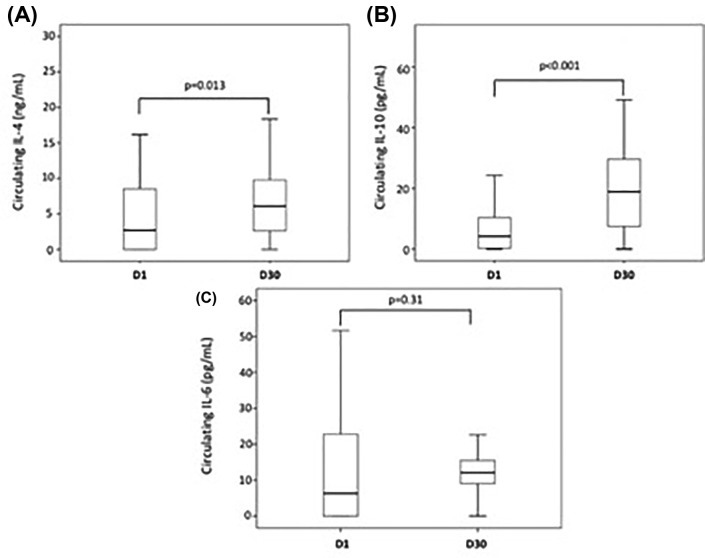
Circulating interleukins after myocardial infarction Box-plots of circulating IL-4 (*n*=87) (**A**), IL-10 (*n*=90) (**B**) and IL-6 (*n*=91) (**C**) at the first (D1) and 30th (D30) days post MI. Titers of circulating IL-4 at D30 were higher compared to D1 (*P*=0.013). Higher titers of IL-10 were seen at D30 compared with D1 (*P*<0.001). Titers of IL-6 did not change at D30 compared with D1 (*P*=0.31). All analyses were made by Wilcoxon test.

There was an increase in the titers of IL-4 and IL-10 at D30 after myocardial infarction compared to D1 (*P*=0.013 and *P*<0.001, respectively, Wilcoxon test), while IL-6 titers did not change (*P*=0.31, Wilcoxon test) (Figure 5). In the supernatant of cultured T lymphocytes, we observed an increase in the titers of IL-6 from samples collected at D30 compared with D1 (*P*=0.028, Wilcoxon test).

At D30, there were correlations between the percentage of B1 lymphocytes with plasma IL-4 titers (rho = 0.24, *P*=0.02) and plasma IL-10 titers (rho = 0.27, *P*=0.01). At D30, negative correlations were observed between the percentage of B2 lymphocytes and IL-10 (rho = −0.26, *P*=0.01) and between the percentage of CD4+ T lymphocytes (%) with IL-4 (rho = −0.26, *P*=0.01). At D30, negative correlation was also observed between B1 lymphocytes (cells/ml) and plasma IL-6 titers (rho = −0.22, *P*=0.04).

### Cardiac MRI, lymphocytes subpopulations and interleukin 6

Main parameters of cardiac MRI are showed in Table 3. The CD11b+ B1 cells at D1 were related to the percentage of infarcted mass rho = 0.43; *P*=0.033 (cells/ml) measured by cardiac MRI at D30.

**Table 3:** Cardiac magnetic resonance imaging parameters

Parameters	*N*=120
Infarcted mass, grams	12.75 (6.35–25.00)
Infarcted mass, %	13.50 (7.05–22.00)
Left ventricular mass, grams	98.00 (78.50–119.00)
Left ventricular ejection fraction, %	52.00 (41.00–60.00)
Right ventricular ejection fraction, %	56.00 (50.00–64.45)

Values are median (IQR) obtained at D30 after STEMI.

**Figure 6:**
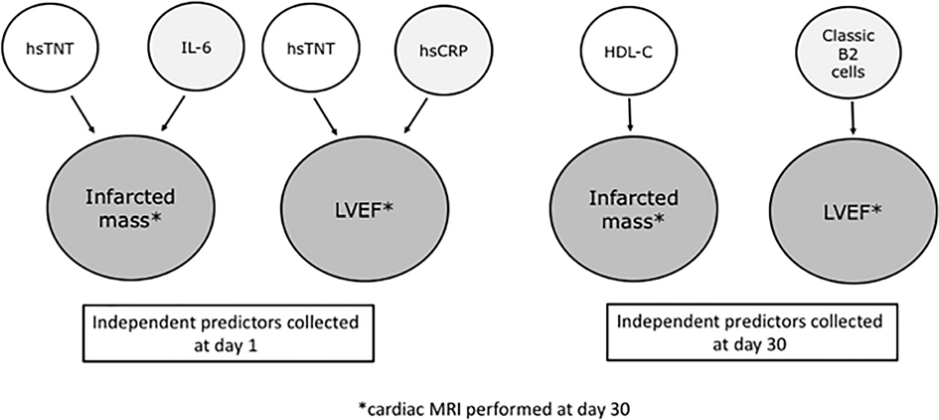
Independent predictors of cardiac magnetic resonance imaging (cMRI) parameters associated with B or T lymphocytes and interleukins Multiple linear regression analysis revealed that circulating titers of high-sensitivity troponin T (hsTNT) at day 1 were independent predictors for the infarcted mass and left ventricular ejection fraction (LVEF) observed by cMRI at day 30. Circulating titers of IL-6 collected at day 1 were also related to the infarcted mass at day 30, while titers of hsCRP collected at day 1 were independent predictors of LVEF at day 30. After 30 days, HDL-C was identified as independent predictor of infarcted mass, while classic B2 lymphocytes (B memory plus B naive cells) collected at day 30 were independent predictors of LVEF at day 30. cardiac MRI, cardiac magnetic resonance imaging; hsCRP, high-sensitivity C-reactive protein; hsTNT, high-sensitivity troponin T; LVEF, left ventricular ejection fraction.

Among interleukins examined at D1, IL-6 titers were associated with the infarcted mass (rho = 0.41, *P*<0.001) and also inversely related to the LVEF estimated by cardiac MRI (rho = -0.38; *P*<0.001). Figure 6 shows the interactions between lymphocyte subtypes with parameters of cardiac MRI.

### Multiple linear regression analysis

Univariate analysis using data collected at day 1 revealed significant associations between infarcted mass (grams) and the following variables: high-sensitivity troponin T (hsTNT), systolic blood pressure, glucose, high-sensitivity C-reactive protein (hsCRP), B1 CD11b+ (%), B1 CD11b+ (cells/ml) and IL-6. In the multiple linear regression analysis (ANOVA F 28.867, *P*<0.001, R square 0.407), hsTNT (beta 0.614, *P*<0.001), and IL-6 (beta 0.256, *P*=0.003) were the independent predictors. Similar results were found for the infarcted mass in percentage of left ventricular mass (data not shown).

Univariate analysis performed with data collected at day 30, showed significant associations between infarcted mass (grams) with the following variables: white blood cells (WBC), HDL-C, serum creatinine, B2 naïve lymphocytes (%), B2 naïve lymphocytes (cels/ml), B2 naïve TLR4+ lymphocytes (%), B2 naive TLR4+ lymphocytes (cells/ml). After multiple linear regression analysis (ANOVA F 10.905, *P*=0.001, R square 0.103), only HDL-C remained independent predictor (beta −0.321, *P*=0.001). The results were similar for the infarcted mass in percentage (data not shown).

Left ventricular ejection fraction (LVEF) obtained at day 30 by cardiac MRI was associated with the following variables collected at day 1 in univariate analysis: hsTNT, total lymphocytes, glucose, hsCRP, and IL-6. After multiple linear regression analysis (ANOVA F 6.268, p = 0.003, R square 0.148), hsTNT (beta −0.286, *P*=0.010), and hsCRP (beta −0.249, *P*=0.025) remained as independent predictors.

The following variables colleted at day 30 were associated with LVEF in univariate analysis: B2 memory lymphocytes (cells/ml), B2 naïve lymphocytes (cells/ml) and B2 classic lymphocytes (cells/ml). After multiple linear regression analysis, B2 classic lymphocytes (cells/ml) remained independent predictors of LVEF (ANOVA F 8.105, *P*=0.005, R square 0.075, beta 0.274, *P*=0.005).

## Discussion

Our study highlights the relevance of B lymphocytes with myocardial infarcted mass and LVEF examined by cardiac MRI 30 days after STEMI. Our findings suggest a detrimental role of CD11b+ B1 cells and circulating IL-6 titers, even after successful coronary reperfusion. However, after multiple linear regression analysis, including clinical and laboratory parameters collected at day 1, the independent predictors for infarcted mass observed by cMRI were hsTNT and IL-6. Regarding the LVEF, multiple regression analysis with variables collected at day 1 showed hsTNT and hsCRP as the independent predictors. In addition, multiple linear regression analysis performed with data collected at day 30 revealed classic B2 lymphocytes as independent predictors of LVEF. There was an association between CD11b+ B1 cells and CD4+ T lymphocytes, a crucial effector cell that can be activated after antigen presentation by B cells, increasing the cytokines released. We also found an increase in the titers of IL-6 in the supernatant of cultured T lymphocytes at D30 compared with D1, which can explain why the IL-6 levels did not decrease at D30, despite significant decrease in the absolute number of lymphocytes, including CD4+ T lymphocytes.

After 30 days of STEMI, there was a shift to anti-inflammatory profile mediated by protective cytokines and, again, B1 lymphocytes seem to be involved due to their positive correlation with IL-4, IL-10, and IgM release. Taken together, B1 lymphocytes appear to play important role in the early stages of inflammatory responses after acute myocardial infarction; however, differences were observed between CD11b+ and CD11b- subsets of B1 lymphocytes. The more protective response seems mediated by CD11b- B1 lymphocytes, while CD11b+ B1 lymphocytes at D1 were related to proinflammatory response, affecting infarcted mass.

Our findings suggest a residual inflammatory status affecting the healing process and ventricular remodeling after coronary reperfusion. All patients in our study were thrombolyzed in the first 6 h of STEMI, received dual antiplatelet therapy and highly effective lipid lowering therapy, performed coronary angiography and underwent percutaneous intervention when needed. However, this standard of care strategy seems insufficient to avoid residual effects of inflammatory cells during myocardial injury repair. In the CANTOS trial [13], subjects with previous myocardial infarction and higher baseline levels of IL-6 presented higher rates of cardiovascular events. Those patients treated by canakinumab, achieving IL-6 levels below the median, had substantial reduction in mortality and cardiovascular events. In addition, in the PLATO study, higher incidence for the composite outcome of cardiovascular death and myocardial infarction was observed among subjects in the highest quartile of baseline IL-6 [14]. Interestingly, IL-6 seems to be a driver for proinflammatory responses related to the impairment of microcirculation [15], affecting the recovery of ischemic tissue post myocardial infarction. Recently, IL-6 was identified as one of the strongest biomarker for infarcted size and LVEF among 131 inflammatory and cardiac biomarkers in subjects after STEMI [12].

In our study, increased titers of IL-4 and IL-10 should be considered a favorable response. In fact, IL-4 may induce a differentiation into less inflammatory phenotypes of T cells and macrophages, and seems beneficial for tissue repair and improvement of ventricular function [16]. In addition, IL-10 can also modify the macrophage phenotype into less inflammatory cell (M2), and has protective role in ventricular remodeling after experimentally induced myocardial infarction [17].

B lymphocytes may present antigens to T lymphocytes triggering proinflammatory responses. In our study CD11b+ B1 cells were related to CD4+ T lymphocytes, an interaction previously reported [6].

The natural antibody release (IgM) by B cells seems protective in experimental models of atherosclerosis [18–21]. In our study, spontaneous release of IgM was observed in cultured B1 cells.

Finally, despite the increase in the protective IL-4 and IL-10 titers 30 days after myocardial infarction, the proinflammatory IL-6 titers were unchanged, and related to the infarcted mass, and lower left ventricular ejection fraction, suggesting the need of additional therapy to improve ventricular remodeling and tissue repair, even after a successful coronary reperfusion. Moreover, circulating IL-6 has been associated with recurrent events and mortality after acute myocardial infarction [13,22].

## Limitations

Inflammatory and immune responses are complex and more comprehensive panel of biomarkers might be necessary for the evaluation of residual inflammatory risk [23,24]. However, our results are innovative, reporting, in humans, a relevant role of subtypes of B lymphocytes in the set of MI patients.

## Conclusions

In subjects with acute myocardial infarction, despite early reperfusion, the amount of infarcted mass and ventricular performance were related to inflammatory responses triggered by circulating B lymphocytes.

## Data Availability

The authors agree to provide study data upon request.

## Abbreviations

DBP: diastolic blood pressure
GFR: glomerular filtration rate
hsCRP: high-sensitivity C-Reactive protein
hs-TNT: high-sensitivity troponin T
LVEF: left ventricular ejection fraction
MI: myocardial infarction
SBP: systolic blood pressure
STEMI: ST segment elevation myocardial infarction
WBC: white blood cells
